# Awareness of Lifestyle and Colorectal Cancer Risk: Findings from the BeWEL Study

**DOI:** 10.1155/2015/871613

**Published:** 2015-10-04

**Authors:** Annie S. Anderson, Stephen Caswell, Maureen Macleod, Angela M. Craigie, Martine Stead, Robert J. C. Steele, The BeWEL Team

**Affiliations:** ^1^Centre for Research into Cancer Prevention and Screening, Cancer Division, Medical Research Institute, Level 7, Ninewells Medical School, Dundee DD1 9SY, UK; ^2^Institute for Social Marketing, University of Stirling, Stirling FK9 4LA, UK; ^3^University of Dundee, Nethergate, Dundee DD1 4HN, UK

## Abstract

It is estimated that 47% of colorectal cancers (CRC) could be prevented by appropriate lifestyles. This study aimed to identify awareness of the causes of CRC in patients who had been diagnosed with a colorectal adenoma through the Scottish Bowel Screening Programme and subsequently enrolled in an intervention trial (using diet and physical activity education and behavioural change techniques) (BeWEL). At baseline and 12-month follow-up, participants answered an open-ended question on factors influencing CRC development. Of the 329 participants at baseline, 40 (12%) reported that they did not know any risk factors and 36 (11%) failed to identify specific factors related to diet and activity. From a potential knowledge score of 1 to 6, the mean score was 1.5 (SD 1.1, range 0 to 5) with no difference between intervention and control groups. At follow-up, the intervention group had a significantly greater knowledge score and better weight loss, diet, and physical activity measures than the control group. Awareness of relevant lifestyle factors for CRC remains low in people at increased risk of the disease. Opportunities within routine NHS screening to aid the capability (including knowledge of risk factors) of individuals to make behavioural changes to reduce CRC risk deserve exploration.

## 1. Introduction

Despite significant advances in our understanding of prevention and early detection, colorectal cancer remains the third most common cancer and cause of cancer death in the UK [1]. Most cases (95%) occur in older adults (>50 years) who commonly have other lifestyle-related conditions including type two diabetes mellitus and cardiovascular disease [2, 3]. These diseases share common risk factors related to obesity, altered glucose-insulin pathways, and abnormal lipids [4, 5]. Meta-analysis studies have demonstrated a consistent association between obesity and CRC (notably in men) and with colorectal adenomas in men and women [6, 7].

Recent UK estimates on cancer preventability indicate that 12% of colorectal cancers could be prevented by increased physical activity, 14% by the avoidance of excess weight, 27% by changes in diet (increasing fibre intake and decreasing red and processed meat), and 7% by reducing alcohol intake [8]. Thus a number of modifiable risk factors can be identified and acted upon for potential reduction of colorectal cancer risk and proven benefit on risk reduction for type two diabetes mellitus and cardiovascular disease.

Investigations in the UK suggest that awareness of lifestyle factors for disease prevention is generally low and is lower for cancer than for heart disease [9]. A recent YouGOV poll commissioned by the World Cancer Research Fund [10] in the UK reported that 59% of people did not know about the links between cancer and body weight and 62% did not know about the association with processed meat. In a secondary data analysis of the US National Health Interview Survey, Bittner Fagan et al. [11] reported that, compared with normal weight respondents, overweight or obese participants did not perceive themselves to be at an increased risk for cancer or specifically for colorectal cancer. Previous qualitative research by our research group [12] reported that people who had been diagnosed with adenomas gave little thought as to what might have caused the adenoma, and in those who gave possible explanations, these tended to relate to age, genetics or “chance.” Similar findings have been reported from studies of cancer survivors where genetic factors, smoking and environmental factors (e.g., pollutants or occupation), and psychosocial factors are the most frequently quoted causes of cancer [13].

The NHS colorectal cancer screening programmes offer a timely opportunity to provide risk factor advice to adults at increased risk of CRC as part of a portfolio of advice (within wider scale population level actions). The current study aims to identify awareness of the causes of CRC in patients who were diagnosed with a colorectal adenoma through the NHS Scotland Bowel Screening Programme and had subsequently been enrolled in a randomised controlled trial of a lifestyle intervention [14].

## 2. Methods

The BeWEL trial was a multicentre randomised controlled trial of a 12-month weight loss intervention delivered by a lifestyle counsellor versus usual care (booklet only) [15]. The intervention was delivered by a lifestyle counsellor who provided a personalised energy prescription with detailed educational information on food choices and a pedometer based physical activity programme as well as body weight scales. Motivational interviewing techniques and behavioural strategies were used to promote relevant changes in diet, physical activity, and body weight [16].

Individuals (aged 50 to 74 years) who had received an adenoma diagnosis following a positive faecal occult blood test and colonoscopy, undertaken through the Scottish Bowel Screening Programme, and who had a BMI ≥ 25 kg/m^2^ were invited to participate in the BeWEL trial. All participants received a letter from an NHS consultant with their adenoma results, endorsing the study and highlighting the importance of lifestyle in adenoma recurrence and CRC risk. A full invitation letter as well as participant information leaflet was then sent out by a research nurse to eligible respondents.

At baseline and 12-month follow-up, questionnaires were administered by the research nurse. Knowledge of lifestyle risk factors for CRC was assessed at both time-points. Participants responded to the open text question “What do you personally think are the main factors that might increase or decrease a person's chance of developing colorectal cancer?” The research nurse encouraged participants to list as many risk factors as possible under the heading of increase risk or decrease risk without prompting or offering any suggestions. Responses were recorded* verbatim* and subsequently scored by the research team. Responses were scored +1 each for body fatness, alcohol, red meat, and processed meat. Foods high in fibre scored +1 if they specified fibre or +0.5 for fruits and vegetables and/or cereals/whole grains/pulses (maximum score +1). The scoring was designed to indicate awareness of all fibre sources rather than fruit and vegetables per se.

Physical activity scored +1 for specifying being physically active/exercise, or +0.5 for sedentary activity (maximum score +1). The total possible score for knowledge of risk factors was +6.

### 2.1. Statistical Analysis

Statistical analyses were carried out using IBM SPSS Statistics for Windows (IBM Corp.: version 21.0, released 2012, Armonk, NY). Descriptive statistics were used to characterise the cohort with regard to knowledge of risk factors for colorectal cancer. Chi-squared tests were performed for comparison of proportions and independent* t*-tests for differences in means. Significance was taken as *p* < 0.05. Between-group differences are presented as odds ratios for differences in proportions, or as mean differences with 95% confidence intervals.

## 3. Results

Full sociodemographic and clinical details of participants at baseline and 12-month follow-up have been presented elsewhere [14]. Participants had a mean age of 63.6 years (SD 6.8) at baseline, with the majority being male (74%) and having had at least some further, professional, or higher education beyond secondary school (60%).

Of the 329 participants, 40 (12%) reported that they did not know any risk factors and a further 36 (11%) failed to identify specific factors related to diet and activity. The mean score for knowledge was 1.5 (SD 1.1, range 0 to 5). The most frequently cited factors were physical activity and alcohol followed by foods containing dietary fibre. Body weight (in this overweight cohort) was cited by 13%. Two participants cited quantitative recommendations (“5 a day” and “2 alcohol free days per week”). It is notable that “low fluid intake” was reported by 8%, suggesting that people were more familiar with this myth than evidence based recommendations to decrease processed meat (6%). Other factors frequently reported included family history (9%) and stress (6%). A number of other risk factors were also cited including bowel function (constipation), sexual transmission, and intake of dairy foods.

No differences in awareness were found between intervention and control groups at baseline; however at 12-month follow-up the intervention group participants were significantly more aware of body fatness (OR 1.99 (95% CI 1.07–3.70)) and red meat (OR 1.99 (95% CI 1.04–3.81)) as CRC risk factors and had a significantly greater mean knowledge score (1.8 versus 1.5, mean difference 0.29 (95% CI 0.05–0.54)) (Table 1). Overall, the results from the main trial showed that the intervention group had significantly better weight loss, diet, and physical activity measures than the control group after 12 months of follow-up [14].

## 4. Discussion

This study aimed to examine the awareness of lifestyle risk factors associated with colorectal cancer risk amongst a cohort of overweight people who had experienced NHS bowel screening and had a diagnosis of an adenoma. Despite these health service experiences in this motivated, high risk group, the results show that the baseline knowledge about CRC risk factors was low.

The study is limited by the use of a selected group of patients who have chosen to participate in a lifestyle trial and may not be representative of the all patients with colorectal adenomas. However, the results concur with previous qualitative research [12] with patients who had had adenoma diagnosis and surgery suggesting that patients are given little information about the potential causes of adenoma during their treatment and that the “all clear” message received after adenoma removal may be interpreted by some patients as meaning that their lifestyle is not a cause of concern.

The findings are consistent with those of Dowswell et al. [17], who studied attitudes to lifestyle in patients aged 60 to 74 years, diagnosed with an intermediate- or high-risk adenoma. They reported that participants believed that their current dietary and physical activity behaviours were good and they perceived no risk between current health behaviours and their adenoma diagnosis. The authors concluded that intervention programmes should tailor interventions to individual habits as well as lack of knowledge about the aetiology of colon cancer. The BeWEL study [14] did target both of these approaches and the intervention was associated with overall change in knowledge and subsequent change in diet and body weight.

It is unlikely that increasing knowledge and awareness of lifestyle and CRC risk factors will in itself promote behaviour change [18], but both are important prerequisites and platforms from which to develop evidence based behavioural change techniques for planning health improvements. The role of knowledge on behaviour change is considered by Michie et al. [19] in the Capability, Opportunity, and Motivation- (COM-) Behaviour (B) model whereby knowledge and skills are two of the factors which can influence the capability of an individual to change health behaviours. This model demonstrates both the importance of individual level influence and how these might be linked to wider public policy.

The NHS CRC screening setting is a unique opportunity to raise awareness about lifestyle and prevention and to provide further guidance and personalised support to enhance the translation of improved knowledge into action. The current work has utilised a one-to-one lifestyle programme to achieve changes in knowledge and health behaviour which may not be routinely possible within NHS budgets but the results strongly support the need to explore the development of lifestyle counsellors in the same way that many health boards employ smoking cessation counsellors.

## 5. Conclusion

Despite a growing evidence base on lifestyle and CRC, awareness of relevant factors remains low in people at increased risk. Opportunities within the routine NHS screening setting to aid the capability of individuals to make effective behavioural changes to reduce CRC risk deserve further exploration.

## Trial Registration

The trial was registered with Current Controlled Trials (ISRCTN53033856).

## Figures and Tables

**Table 1:** Knowledge of risk factors for colorectal cancer.

	All	Intervention		Control		Between-group difference at baseline OR/mean (95% CI) *p* value	Between-group difference at 12 months follow-up OR/mean (95% CI) *p* value
	Baseline *n* = 329	Baseline *n* = 163	12-month follow-up *n* = 148	Baseline *n* = 166	12-month follow-up *n* = 156		
Alcohol (%)	148 (45.0)	73 (44.8)	77 (52.0)	75 (45.2)	76 (48.7)	0.98 (0.64–1.52) 0.943	1.14 (0.73–1.79) 0.564

Physical activity (%)	153 (46.5)	81 (49.7)	86 (58.1)	72 (43.4)	80 (51.3)	1.29 (0.84–1.99) 0.251	1.32 (0.84–2.07) 0.232

Body fatness (%)	44 (13.4)	25 (15.3)	32 (21.6)	19 (11.4)	19 (12.2)	1.40 (0.74–2.66) 0.300	1.99 (1.07–3.70)^*∗*^ 0.028

Foods containing fibre (%)	111 (33.7)	57 (35.0)	58 (39.1)	54 (32.6)	56 (35.9)	1.12 (0.71–1.76) 0.640	1.15 (0.73–1.83) 0.553

Red meat (%)	44 (13.4)	21 (12.9)	29 (19.6)	23 (13.9)	17 (10.9)	0.92 (0.49–1.74) 0.796	1.99 (1.04–3.81)^*∗*^ 0.034

Processed meat (%)	6 (1.8)	2 (1.2)	3 (2.0)	4 (2.4)	5 (3.2)	0.50 (0.09–2.79) 0.423	0.63 (0.15–2.66) 0.521

Quantity mentioned (%)	2 (0.6)	1 (0.6)	1 (0.7)	1 (0.6)	0 (0)	1.02 (0.06–16.42) 0.990	~ 0.304

Do not know any (%)	40 (12.2)	15 (9.2)	12 (8.1)	25 (15.1)	15 (9.6)	0.57 (0.29–1.13) 0.104	0.83 (0.38–1.84) 0.644

Total score [mean (SD)] Range	1.5 (1.12) 0–5	1.5 (1.07) 0–5	1.8 (1.06) 0–5	1.4 (1.17) 0–5	1.5 (1.11) 0–5	0.12 (−0.36 to 0.13) 0.340	0.29 (0.05 to 0.54)^*∗*^ 0.019

^*∗*^ *p* < 0.05, significant.
